# Overnight affective dynamics and sleep characteristics as predictors of depression and its development

**DOI:** 10.1192/j.eurpsy.2021.877

**Published:** 2021-08-13

**Authors:** O. Minaeva, S. George, A. Kuranova, M. Wichers, H. Riese, S. Booij

**Affiliations:** Department Of Psychiatry, University of Groningen, University Medical Center Groningen, Groningen, Netherlands

**Keywords:** Affective inertia, Depression, sleep, Experience Sampling Method

## Abstract

**Introduction:**

Greater affective inertia during the day (higher carry-over effects of prior affect to the current moment) is associated with depression and its development. However, the role of overnight affective inertia (from evening to morning) in depression, and the role of sleep therein, has been scarcely studied.

**Objectives:**

We examined i) the difference in overnight inertia for positive (PA) and negative affect (NA) between individuals with past depression, current depression, and no depression; ii) how sleep duration and quality influence overnight affective inertia in these groups, and iii) whether overnight affective inertia predicts depression development.

**Methods:**

We used data of 579 women from the East-Flanders Prospective Twin Survey. First, individuals with past (n=82), current (n=26), and no depression (n=471) at baseline were examined, and then individuals who did (n=58) and did not (n=319) develop depression at 12-months follow-up. Affect was assessed 10 times a day for 5 days. Sleep was assessed with sleep diaries. Affective inertia was operationalized as the influence of affect_t-1_ on affect_t_. Linear mixed-effect models were used to test the hypotheses.

**Results:**

Overnight affective inertia was not associated with depression, neither was it differently associated with sleep characteristics in the depression groups. However, sleep characteristics were more negatively associated with morning NA in both depression groups compared to the non-depressed group. Overnight affective inertia did not predict the development of depression at follow-up.

**Conclusions:**

Depression and sleep characteristics might be more related to mean affect levels rather than to more complex emotion dynamics measures. Replication of these findings with longer time-series is needed.

